# Pruritus in Lichen Planopilaris and Frontal Fibrosing Alopecia—Clinical Characteristics and Dermoscopic Correlations

**DOI:** 10.3390/jcm13164898

**Published:** 2024-08-19

**Authors:** Kinga Kołcz, Karolina Krawczyk-Wołoszyn, Adam Reich, Magdalena Żychowska

**Affiliations:** 1 Department of Dermatology, Institute of Medical Sciences, Medical College of Rzeszow University, 35-959 Rzeszow, Poland; kingakolcz@gmail.com (K.K.); karolinakrawczyk10@wp.pl (K.K.-W.); adi_medicalis@go2.pl (A.R.); 2 The Doctoral School, University of Rzeszow, 35-959 Rzeszów, Poland

**Keywords:** itch, pruritus, scarring alopecia, lichen planopilaris, frontal fibrosing alopecia

## Abstract

**Background:** Lichen planopilaris (LPP) and frontal fibrosing alopecia (FFA) are the most common causes of lymphocytic scarring alopecia. Itching of the scalp is a common accompanying symptom. The aim of the study was the clinical assessment of pruritus and its correlation with dermoscopic features. **Methods:** Sixty-one patients with scarring alopecia were analyzed (LPP = 16; FFA = 33; coexisting LPP-FFA = 12). Each patient underwent a trichoscopic examination. Itch severity and characteristics were assessed using a Visual Analogue Scale (VAS), 4-item Itch Questionnaire and 12-item Descriptive Pruritus Assessment Questionnaire. **Results:** Itching of the scalp occurred in 73.8% of the patients (mean maximal VAS 5.3 ± 3.1 points). Pruritus was most frequently accompanied by tingling (19.7%) or burning (14.8%) sensations. The following factors most frequently increased the severity of pruritus: sweating, heat, stress and hot water. On the other hand, cold water and cold air often relieved symptoms. There was a significant relationship between itch and perifollicular scaling (*p* = 0.011), hair diameter diversity (*p* = 0.008) and white halo (*p* = 0.016). **Conclusions:** Pruritus was the main subjective complaint reported by patients suffering from LPP and FFA. A better understanding of pruritic features may help in the selection of an effective therapeutic strategy.

## 1. Introduction

Lichen planopilaris (LPP) is one of the subtypes of primary scarring lymphocytic alopecias [1]. It was first described by Pringle in 1895 [2]. The etiopathogenesis of the disease is not yet fully understood; however, it is suggested to be related to the autoimmune reaction of T lymphocytes to an unknown antigen in the hair follicle, leading to the destruction of the follicle’s multipotent stem cells and the formation of scar tissue [3]. LPP is considered by some authors to be a variant of lichen planus (LP). Nevertheless, only about 30% of patients present with eruptions on non-hairy skin or mucous membranes in addition to scalp involvement [4]. LPP affects women, predominantly between 30 and 70 years of age, more often than men [5,6]. Based on the distribution of scalp lesions, three clinical forms of LPP have been initially distinguished in the literature. The classical variant of LPP is characterized by foci of scarring alopecia that are most commonly located on the vertex and parietal regions but may also be distributed throughout the scalp. On physical examination, perifollicular erythema with scaling is typically present. The lesions are usually accompanied by pruritus of the scalp [7]. Graham-Little-Piccardi-Lassueur syndrome (GLPLS) is a rare subtype of LPP, in which, in addition to foci of scarring alopecia on the scalp, non-scarring loss of axillary and pubic hair coexist with follicular papules on the glabrous skin of the trunk and extremities [8]. In 2000, Zinkernagel and Trüeb reported a case series of progressive scarring alopecia with the presence of lymphocytic infiltration in histopathology, which occurred in areas typically involved in androgenic alopecia (AGA) or female pattern hair loss. Considering the overlapping clinical and histopathological features, the term Fibrosing Alopecia in a Pattern Distribution (FAPD) was coined. The condition has been classified as another variant of LPP [9].

Frontal fibrosing alopecia (FFA) was first described by Kossard in 1994 as a subtype of lymphocytic scarring alopecia characterized by receding hairlines in the frontal and temporoparietal regions and was initially referred to as postmenopausal frontal fibrosing alopecia [10]. In 1997, the same author referred to it as the anterior variant of LPP [11]. FFA is often accompanied by loss of the eyebrows, especially the outer portions [12]. FFA predominantly affects postmenopausal women, but there is an increasing number of case reports in younger women and even men [13]. The clinical picture of perifollicular lesions, perifollicular exfoliation and perifollicular erythema is very similar to the classic form of LPP; moreover, the histopathological changes also show great similarity [14]. Despite the significant histopathological similarity, the link between FFA and LPP is disputed by many authors. Currently, FFA is considered the most common subtype of scarring alopecia [15]. In view of the much higher incidence of FFA in the peri- or postmenopausal period, many authors suggest a link with a sudden decrease in estrogen levels, which may be reflected in an accelerated catagen to telogen transition in the hair growth cycle. Hormonal imbalance may induce the inflammatory response with subsequent fibrosis of the hair follicle region and development of scarring alopecia [16,17]. In addition, FFA can also take on atypical clinical forms, such as the AGA-like pattern, in which symmetric frontal-temporal hairline recession with an unchanged paramedian frontal line is observed [18]. Katoulis et al. raised the hypothesis that FFA and AGA may be pathogenetically related, and even FFA may represent a scarring variant of AGA. This theory is supported by the clinical improvement of AGA, FFA and FAPD after implementation of aromatase inhibitors [19]. This further underscores the potential role of androgens in the development of an inflammatory response resulting in scarring in an immunogenetically predisposed individual [20]. It is also worth mentioning that regardless of the nosological positions of FFA and LPP, the two entities can coexist as LPP-FFA. Such a case series has been described by Saceda-Corralo et al., and the authors emphasized that although both diseases are lymphocytic scarring alopecia, their coexistence can lead to the misdiagnosis of one or the other [21].

Regardless of the classification of the aforementioned conditions, according to the literature, pruritus of the affected area is the most frequently reported subjective symptom in all clinical forms of LPP [3,6,7]. It can alter a patient’s mood and, if chronically present, it can significantly reduce quality of life and even cause depression or anxiety [22,23]. Studies carried out so far showed that pruritus can affect up to 70% of patients with LPP and 67% of patients with FFA [22]. Despite such high prevalence, the clinical aspects of pruritus have not been characterized yet. The aim of this study was to analyze the severity and clinical characteristics of this unpleasant sensation in patients with LPP in relation to dermoscopic findings.

## 2. Materials and Methods

### 2.1. Study Population

Consecutive patients with classical variants of LPP and FFA referred to the Department of Dermatology in Rzeszow between January 2021 and April 2024, were recruited to the study. The diagnosis was based on clinical and trichoscopic presentation and confirmed by histopathology in every doubtful case. A specially designed questionnaire was used to collect demographic (gender, age) and clinical data (e.g., age at which first symptoms occurred, duration of the disease, duration of current exacerbation, prior treatment, presence of comorbidities, etc.). Patients with other dermatological conditions that could affect the assessment, as well as those undergoing treatment for LPP/FFA, were excluded from the study.

**Figure 1:**
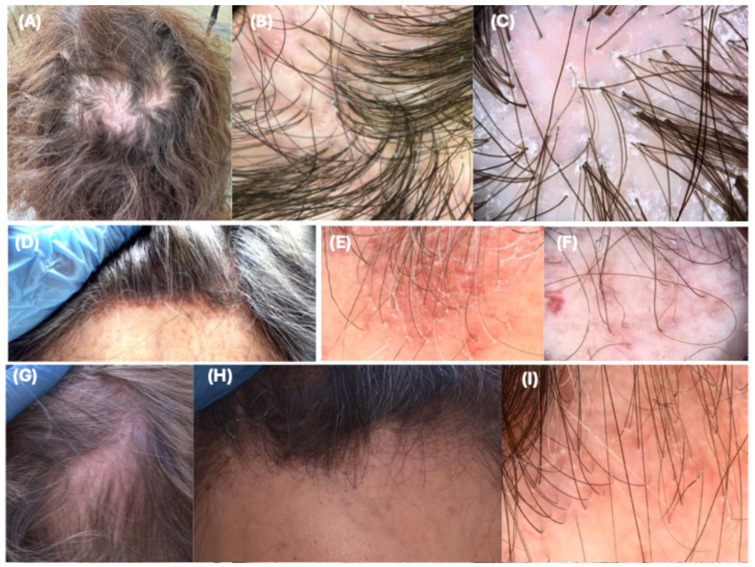
Clinical and dermoscopic presentation of patients from each study group: (**A**)—Clinical view of patient with lichenplanopilaris (LPP), (**B**)—Dermoscopy (DermLite DL5, *San Juan* Capistrano, CA, USA, 10× magnification) performed in the parietal lesion in LPP, (**C**)—Videodermoscopy (Canfield D200^EVO^, Parsippany, NJ, USA) performed in the parietal lesion in LPP, (**D**)—Clinical view of patient with frontal fibrosing alopecia (FFA), (**E**,**F**)—Videodermoscopy (Canfield D200^EVO^, Parsippany, NJ, USA) performed in the frontal hairline of FFA, (**G**,**H**)—clinical presentation of patient with co-occurrence LPP—FFA, (**I**)—Videodermoscopy (Canfield D200^EVO^, Parsippany, NJ, USA) performed in the frontal hair line of LPP-FFA.

In total, 61 patients (53 women and 8 men with a mean age ± standard deviation (SD) of 58.8 ± 12.9 years) were recruited. Based on the clinical presentation, three groups were subsequently distinguished: (1) patients with classical variant of LPP involving predominantly the vertex and parietal scalp, referred to as LPP (n = 16); (2) patients with typical presentation of FFA comprising frontal and/or temporal recession of the hairline, referred to as FFA (n = 33); (3) patients displaying clinical features of both aforementioned subtypes, referred to as LPP-FFA (n = 12)—Figure 1.

The study was approved by the Bioethics Committee of University of Rzeszow (Decision no 2023/04/0023) and was conducted according to the Declaration of Helsinki. All participants signed a written informed consent form prior to any procedure.

### 2.2. Assessment of Itch

Itch severity was assessed using 2 different methods: the 10 cm visual analogue scale (VAS) and the 4-item itch questionnaire containing questions about number of itch locations (1 location—1 point; several locations—2 points; generalized scalp pruritus—3 points), the necessity for scratching (no need—1 point; need for scratching to obtain relieve—2 points; scratching giving no relieve—3 points; presence of excoriations—4 points; complete irritation caused by itching—5 points); number and duration of itch episodes during the day (four shorter episodes—1 point each; one longer episode—1 point each; persistent pruritus—5 points) and sleep disturbances (each episode—2 points; maximum 6 points). The total score of the 4-item questionnaire can range from 0 (no itch) to 19 points [24].

For the pruritus measurement using VAS, each patient indicated with a vertical line on a 10 cm scale the severity of itch at the time of examination (VAS_exam_), the maximum severity of itch during the course of the disease (VAS_max_) and, to establish a reference point, the severity of itch after a mosquito bite (VAS_mosquito_) [25,26].

In addition, the clinical characteristics of itch were thoroughly analyzed using Descriptive Pruritus Evaluation Questionnaire, containing questions about the itch severity, frequency and duration, as well as pruritus-induced disturbances of daily activities or sleep, psychological well-being and exacerbating/alleviating factors [27].

### 2.3. Dermoscopy (Trichoscopy)

All study participants underwent non-invasive dermoscopic imaging of the scalp using Canfield D200^EVO^ Videodermatoscope (Parsippany, NJ, USA). At least one image without the use of immersion fluid (for better visualization of the scaling) and at least one image with the use of ultrasound gel as the immersion fluid (for better visualization of the interfollicular surface) was obtained. The images were then stored and subsequently analyzed by the first and last authors (K.K. and M.Ż.) for the presence of trichoscopic findings associated with follicular openings, hair shafts, perifollicular and interfollicular surfaces, and vessels.

### 2.4. Statistical Analysis

Data were analyzed using SPSS. Categorical data were presented as numbers and percentages, and continuous data—as means with standard deviations (SD) of the mean, and medians. Mann–Whitney’s U test and Fisher’s exact test were used for comparisons between the groups. Values of *p* < 0.05 were considered statistically significant.

## 3. Results

### 3.1. Clinical Characteristics of the Study Population

The study included sixty-one adult patients with LPP (n = 16), FFA (n = 33) or co-occurrence of LPP-FFA (n = 12). In 33 of the 61 patients, a biopsy was performed to confirm the diagnosis. Detailed characteristics of the study participants are shown in Table 1.

### 3.2. Frequency and Duration of Itch

Forty-five (73.8%) study participants experienced scalp pruritus during the course of the disease, of which 35 (59.0%) patients reported presence of pruritus during the last 3 days preceding the examination. Sixteen (26.23%) patients denied having scalp pruritus at all. Among the patients with pruritus, the majority experienced itch on a daily basis (n = 22, 48.9%), and a significant group of patients (n = 18, 40.0%) complained of itch at least several times a week, while only five (11.1%) patients had episodes of itching less frequently than once a week. Itching occurred most frequently in the evening (n = 41, 91.1%), while only 17 (37.8%) patients reported having itch episodes at night.

Considering the length of pruritus episodes, nearly equal numbers of patients indicated having episodes shorter than 1 min or lasting between 1 and 10 min (n = 16, 35.6% and n = 15, 33.3%, respectively). Fourteen (31.1%) patients responded that every itch episode lasted longer than 10 min.

### 3.3. Intensity of Itch

Itch severity at the time of examination (VAS_exam_) ranged from 0.1 to 8.9 points (mean 2.4 ± 2.4), while the maximum pruritus intensity (VAS_max_) reported by the patients was from 0.5 to 10 points (mean 5.3 ± 3.1). Interestingly, the intensity of maximum pruritus in the study group was comparable to the itch intensity reported after a mosquito bite (mean 5.7 ± 2.6, *p* = 0.519). The intensity of pruritus assessed with the 4-item Itch Questionnaire ranged from 3 to 15 points (mean 6.4 ± 2.7 points).

### 3.4. Itch-Accompanying Sensations and Sleeping Problems

Study participants were asked about the sensations accompanying pruritus. The patients most frequently reported tingling (n = 12, 26.7%), pain (n = 10, 22.2%) and burning sensation (n = 9, 20.0%). Additionally, equal numbers of patients experienced tickling or pinching sensation (n = 8, 17.8%). The smallest number of patients reported pricking sensation accompanying itch (n = 3, 6.7%). In about one-third of patients, the occurrence of scalp pruritus did not affect their psychological state (n = 17, 37.8%). However, in some patients, scalp itch was a reason for lowered mood (n = 13, 28.9%), and in seven (15.6%) patients it caused problems with concentration. Regarding the influence of scalp pruritus on the quality of sleep, 12 (26.7%) patients had problems falling asleep, 10 (22.2%)—woke up from sleep, and 6 (13.3%)—reached for sleeping pills for this reason.

### 3.5. Factors Influencing Itch

**Figure 2:**
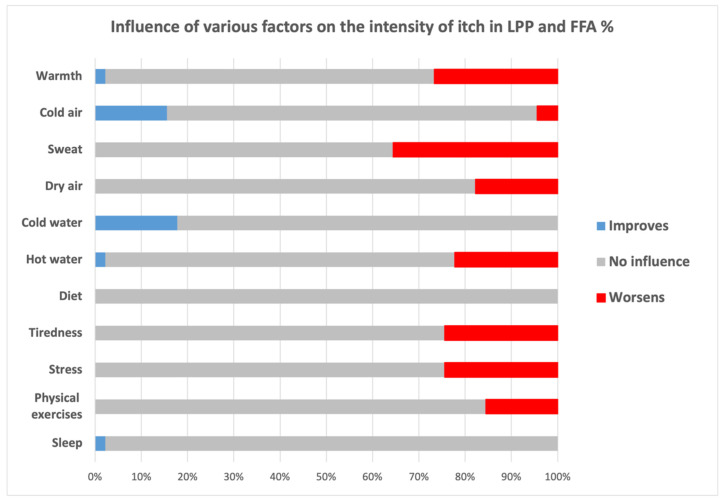
Influence of various factors on the intensity of itch in study participants.

A summary of factors exacerbating and alleviating itch is presented in Figure 2. Major factors exacerbating itching included sweat (55.6%), warmth (51.1%), stress (37.8%), hot water (37.8%) and fatigue (35.6%), while cold water (24.4%) and cold air (20.0%) constituted two main factors reducing itch. None of the respondents observed a correlation of symptoms with diet.

### 3.6. Comparison of Itch Characteristics in Patients with LPP, FFA and LPP-FFA

Twenty-one out of thirty-three patients with FFA experienced scalp pruritus in the course of the disease. The mean itch severity measured with VAS_exam_ was 2.0 ± 2.5 points, while mean VAS_max_ was 4.6 ± 3.2 points. In patients with classical LPP (n = 16), scalp pruritus was present in the vast majority (n = 13; 81.3%), and its mean severity was 2.8 ± 2.6 points at the time of examination (VAS_exam_) and 6.2 ± 2.6 points in the worst of times (VAS_max_). In patients presenting with clinical features of both LPP and FFA (n = 12), itch was present in 11 (91.6%) cases, with mean VAS_exam_ score of 2.8 ± 2.2 points, and mean VAS_max_ of 5.9 ± 3.3 points. No statistically significant differences were found between the patients with classical LPP, FFA and co-occurrence of LPP and FFA in terms of presence of pruritus, itch severity or other clinical features of itch. Details are presented in Table 2.

### 3.7. Correlation of Itch with Dermoscopic Findings

Dermoscopy (trichoscopy) was performed on all study participants. Absence of follicular openings was observed in all (100.0%) cases. The predominant dermoscopic features were the presence of interfollicular white (96.7%) or pink (83.6%) structureless areas, perifollicular erythema (82.0%), perifollicular scaling (70.5%), perifollicular pigmentation (63.9%) and hair diameter diversity (60.7%). The analyzed clinical groups did not differ significantly in terms of dermoscopic presentation, except for perifollicular scaling, which was more frequently observed in LPP and LPP-FFA (*p* = 0.012). Details of dermoscopic findings in study participants are presented in Table 3.

When assessing the relationship between the presence and severity of pruritus and individual dermoscopic findings, a significantly more frequent presence of itch in patients with perifollicular scaling (*p* = 0.011), hair diameter diversity (*p* = 0.008) and white halo (*p* = 0.016) was noted. In addition, patients with yellow dots under trichoscopy reported significantly more severe itch at examination (VAS_exam_)—*p* = 0.020. On the contrary, patients with a honeycomb pigment pattern present in trichoscopic examination complained of significantly less severe pruritus at the time of examination than patients without this trichoscopic feature (*p* = 0.012). Patients with perifollicular scaling also experienced significantly more severe scalp pruritus (VAS_max_) in the course of the disease (*p* = 0.005), while the opposite was observed for patients with a honeycomb pigment pattern (*p* = 0.037). Details are presented in Table 4.

## 4. Discussion

The dispute whether FFA is a variant of LPP, or a separate entity remains unresolved. Clinically, these two entities differ predominantly in the localization of scalp involvement. Harries et al. hypothesized that LPP and FFA represent two phenotypically different branches of the same tree, thus explaining the concept that the two entities share clinical and histologic features of lichenoid infiltration [28]. Poblet et al. tried to find histological features that clearly differentiate FFA and LPP. The authors suggested that FFA is characterized by a milder inflammatory infiltrate, with more prominent necrotic keratinocytes and a greater response to foreign bodies. They also noted that FFA infiltration causes less damage to the basal layer and less damage to the interfollicular epidermis, which could explain the less frequent presence of perifollicular scaling in FFA [29]. It seems that minor histopathological differences may be reflected in the trichoscopic features observed in LPP and FFA [18].

Regardless of the classification, both conditions belong to the group of primary scarring alopecias with lymphocytic infiltration, in which itching of the scalp, in addition to erythema and cuff-like perifollicular scaling, is considered one of the indicators of disease activity [30,31]. Overlapping features of LPP and FFA are not uncommon. Having this in mind, we analyzed the clinical aspects of pruritus both in the entire study group and in the three subgroups distinguished based on the clinical presentations. Studies conducted to date show that up to one-third of FFA patients complain of pruritic skin or trichodynia [32]. In our study, the number of patients reporting pruritus was even twice as high (63.6%). In addition, persistent pruritus is considered to be one of the factors that reduces quality of life. The English-language literature available to date lacks an in-depth characterization of pruritus as one of the clinical manifestations of LPP and/or FFA. Welz-Kubiak et al. analyzed the clinical aspects of pruritus in lichen planus (LP) involving glabrous skin beyond the scalp [23]. Comparing the results of our study to those obtained by Welz-Kubiak et al., it seems that itching in LPP/FFA is less severe than in LP beyond the scalp, as the mean VAS_max_ in LPP/FFA was 5.6 points, and in LP—6.9 points [23]. Apart from LP, the sensation of itching accompanies a wide variety of dermatoses. It is estimated that approximately 22–26% of the general population suffers from itchy skin. This unpleasant sensation most frequently occurs in patients with atopic dermatitis (87%), psoriasis (84%) and those over 65 years of age (78%) [33]. Other subjective symptoms, such as pain or burning, may also be present in the acute phase of LPP/FFA [34,35]. In our study, tingling was the subjective symptom that was more frequently reported by the participants than pain.

Sleep disturbances are observed in many chronic autoimmune diseases [36]. Also in our study, we evaluated the impact of pruritus of the scalp on sleep quality disturbances. The results showed that sleep disturbances in patients with LPP and/or FFA are mainly related to falling asleep and sleep maintenance. Similar conclusions were reached by Park et al., who performed an in-depth assessment of sleep in alopecia areata using the Pittsburgh Sleep Quality Index questionnaire [37]. Sleep quality in different subtypes of psoriasis was assessed by Jaworecka et al. [38], while Adamo et al. [39] analyzed this aspect in oral lichen planus. Both research teams unanimously highlighted the key role of pruritus as a determinant of a patient’s quality of life and sleep quality. The available literature lacks reports directly assessing the impact of pruritus in the course of LPP/FFA on patients’ quality of life. In contrast, Nasimi et al. demonstrated the relationship between impairment in quality of life and mental health in patients with LPP and the degree of disease activity, measured with the LPPAI scale [40].

Factors exacerbating and alleviating scalp pruritus in the course of LPP and/or FFA were also analyzed. In our participants, sweat and physical activity were most frequently mentioned among exacerbating factors. The hypothesis of exacerbation of FFA activity by sweat has previously been considered by Harries et al. [41]. The authors reported a series of 11 women with FFA, who at the same time complained of increased scalp sweating. Harries et al. proposed that neurogenic inflammation and changes in local neuropeptide signaling regulating sweat secretion may be considered the trigger factors for FFA and concomitant itch development [41]. However, the hypothesis requires studies on a larger group of patients. Factors exacerbating and alleviating pruritus identified in our study slightly differ from the results reported by Welz-Kubiak et al. in patients with LP involving glabrous skin [23]. In the aforementioned study, heat and high temperature were reported as the most significant factors triggering itch [23].

According to Rajan et al., perifollicular scaling is observed early in the course of LPP [35]. Furthermore, Lajevardi et al. found a statistically significant correlation between the LPP activity index and the occurrence of cuff-like perifollicular scaling. It is considered the most characteristic symptom of the active phase of LPP [42]. In our study, perifollicular scaling (*p* = 0.011), along with hair diameter diversity (*p* = 0.008) and white halo (*p* = 0.016), significantly more frequently co-occurred with pruritus of the scalp. Rajan et al., in an attempt to differentiate between LPP and FFA based on trichoscopic examination, noted that exfoliation was more severe with LPP [35]. These observations are consistent with the results obtained in our study group of LPP and FFA, in which perifollicular scaling was present in 87.5% and 54.5% of patients, respectively. However, an even greater percentage of perifollicular scaling was demonstrated in patients with coexisting features of LPP and FFA—91.7%.

Toledo-Pastarna et al. linked the presence of perifollicular erythema and scaling with pruritus of the scalp. The authors confirmed that erythema is caused by the lymphocytic lichenoid infiltrate observed on histopathology, and it may be responsible for the occurrence of pruritus and, thus, constitute a marker of FFA activity [31,43]. Fernández-Crehuet et al., in their descriptive, retrospective, observational, multicenter study, based on trichoscopic images of 249 patients, found a statistically significant relationship between the presence of white dots and disease severity. However, neither of the aforementioned studies precisely characterized scalp itch or correlated it with trichoscopic features [31,43]. One of the main findings of our analysis is the more frequent presence of itch in patients showing perifollicular scaling. Patients with perifollicular scaling reported also significantly more severe itch in the course of the disease. Although we also thoroughly assessed the presence of perifollicular erythema, we did not observe any relationship with the occurrence or severity of pruritus in our study participants.

It is also worth noting that, according to our results, itching was more intense not only in patients with perifollicular scaling, but also when yellow dots were present under trichoscopy. Yellow dots, although much more characteristic of AGA, were also reported in early FFA. They are also considered to be indicators of early disease stage when sebaceous glands are preserved, and hair regrowth is potentially possible [44].

Hair diameter diversity is primarily associated with AGA and indeed, loss of vellus hair in the frontal hairline is believed to be one of the most common symptoms of mild FFA. Interestingly, Saceda-Corralo et al. conducted a study in which they showed that vellus hair can remain partially or completely preserved in 25% and 8.9% of patients with FFA, respectively [45]. In addition, a subtype of FFA with a pseudo-fringe sign was distinguished. This subtype of FFA, with preserved vellus hair, is associated with good prognosis [45,46,47]. In our study, hair diameter diversity along with perifollicular scaling, correlated with scalp pruritus (*p* = 0.008). Considering the preservation of vellus hair and presence of yellow dots in mild FFA and the co-occurrence of increased pruritus, we can suspect a very early stage of the disease that could potentially, when treated, result in hair regrowth.

On the contrary, itching was significantly less severe in patients in whom a honeycomb pigment pattern was observed. The honeycomb pigment pattern is a frequently reported trichoscopic finding in long-standing AGA. However, it may be observed in FFA, FAPD or classic LPP, as well [48,49,50]. The markedly less severe pruritus observed in patients with a honeycomb pigment pattern may be related to the long-lasting but slow disease process.

The major limitations of the study included the single-center design and a relatively small group of participants. It should be also taken into consideration, that the assessment of itch intensity and accompanying symptoms is very subjective. To overcome this difficulty, previously validated scales and questionnaires were used.

## 5. Conclusions

In conclusion, we comprehensively characterized the clinical features of scalp pruritus in LPP/FFA. Due to its long-standing course and high incidence, pruritus is a troublesome symptom that further impairs the quality of life of patients with permanent hair loss. A better understanding of this symptom may help in the selection of a more patient-orientated therapeutic strategy.

## Figures and Tables

**Table 1:** Demographic and clinical characteristics of study participants.

Patient Characteristics	Total (n = 61)	LPP (n = 16)	FFA (n = 33)	LPP-FFA (n = 12)
Sex, n (%)				
Women	53 (86.9)	9 (56.3)	32 (97.0)	12 (100.0)
Men	8 (13.1)	7 (43.7)	1 (3.0)	0 (0.0)
Age (years), mean ± SD	58.8 ± 12.9	55.0 ± 14.1	58.6 ± 11.6	64.5 ± 14.1
Range	26–83	26–74	33–83	36–81
Disease duration (months), mean ± SD	76 ± 105.8	40.9 ± 47.3	69.3 ± 95.0	141.2 ± 161.7
Range	4–540	6–180	4–540	11–480
Duration of the current exacerbation (months), mean ± SD	47.6 ± 83.9	32.5 ± 32.3	51.5 ± 95.4	56.8 ± 102.3
Range	0–540	3–120	0–540	2–360

SD: Standard deviation.

**Table 2:** Comparison of the detailed characteristics of pruritus by separated patient groups (ns—nonsignificant; n—number of cases; VAS—visual analogue scale).

Patient Characteristics	Total (n = 61)	LPP (n = 16)	FFA (n = 33)	LPP—FFA (n = 12)	*p* Value
Presence of pruritus, n (%)	45 (73.8)	13 (81.3)	21 (63.6)	11 (91.6)	ns
VAS_exam_ (cm)					ns
mean ± SD	2.4 ± 2.4	2.8 ± 2.6	2.0 ± 2.5	2.8 ± 2.2	
range	0–8.9	0–8.5	0–8.9	0–6.2	
VAS_max_ (cm)					ns
mean ± SD	5.3 ± 3.1	6.2 ± 2.6	4.6 ± 3.2	5.9 ± 3.3	
range	0.5–10	2.3–10	0.1–10	0.7–9.8	
VAS_mosquito_ (cm)					ns
mean ± SD	5.7 ± 2.6	5.9 ± 2.1	5.5 ± 2.7	5.9 ± 3.1	
range	0–10	3.5–9.1	0–10	0.7–9.7	
4-item itch questionnaire (points)					ns
mean ± SD	6.4 ± 2.7	5.8 ± 1.9	6.7 ± 3.1	6.4 ± 2.9	
range	3–15	4–10	4–15	3–12	
Duration of pruritus, n (%)					ns
<1 min	16 (26.2)	5 (31.3)	8 (24.2)	3 (25.0)	
Several minutes	15 (24.6)	4 (25.0)	5 (15.2)	6 (50.0)	
>10 min	14 (23)	4 (25.0)	8 (24.2)	2 (16.7)	
Frequency of pruritus, n (%)					ns
Every day	22 (36.1)	6 (37.5)	10 (30.3)	6 (50.0)	
Several times/week	18 (29.5)	7 (43.8)	7 (21.2)	3 (25.0)	
Several times/month	5 (8.2)	0 (0.0)	4 (12.1)	2 (16.7)	
Less than 1/month	16 (26.2)	3 (18.8)	12 (36.4)	1 (8.3)	
Preparations used to reduce itching, n (%)					ns
Corticosteroids	6 (9.8)	3 (18.8)	2 (6.1)	1 (8.3)	
Emollients	3 (4.9)	1 (6.3)	1 (3.0)	1 (8.3)	
Anti-dandruff shampoos	2 (3.3)	0 (0.0)	1 (3.0)	1 (8.3)	
Time of the day of pruritus, n (%)					ns
Morning	41 (67.2)	12 (75)	19 (57.6)	10 (83.3)	
Afternoon	39 (63.9)	12 (75)	17 (51.5)	10 (83.3)	
Evening	41 (67.2)	11 (68.8)	20 (60.6)	10 83.3)	
Night	17 (27.9)	6 (37.5)	8 (24.2)	3 (25.0)	
Itch-associated sensations, n (%)					ns
Tickling	8 (13.11)	4 (25.0)	3 (9.1)	1 (8.3)	
Pricking	3 (4.92)	0 (0.0)	2 (6.1)	1 (8.3)	
Tingling	12 (19.67)	3 (18.8)	8 (24.2)	1 (8.3)	
Pinching	8 (13.11)	1 (6.3)	4 (12.1)	3 (25.0)	
Burning sensation	9 (14.75)	3 (18.8)	4 (12.1)	2 (16.7)	
Itch perception, n (%)					ns
Burdensome	10 (16.4)	2 (12.5)	5 (15.2)	3 (25.0)	
Irritating	20 (32.8)	7 (43.8)	8 (24.2)	5 (41.7)	
Unbearable	5 (8.2)	2 (12.5)	1 (3.0)	2 (16.7)	
Disturbing	11 (18.0)	2 (12.5)	6 (18.2)	3 (25.0)	
Difficulties falling asleep, n (%)					ns
Nearly always	4 (6.6)	0 (0.0)	3 (9.1)	1 (8.3)	
Rarely	8 (13.1)	5 (31.3)	1 (3.0)	2 (16.7)	
never	32 (52.5)	7 (43.8)	17 (51.5)	8 (66.7)	
Waking up from sleep, n (%)					ns
Nearly always	4 (6.6)	0 (0.0)	3 (9.1)	1 (8.3)	
Rarely	6 (9.8)	2 (12.5)	2 (6.1)	2 (16.7)	
Never	33 (54.1)	9 (56.3)	16 (48.5)	8 (66.7)	
Use of sedatives, n (%)					ns
Nearly always	2 (3.3)	0 (0.0)	2 (6.1)	0 (0.0)	
Rarely	4 (6.8)	1 (6.3)	1 (3.0)	2 (16.7)	
Never	37 (60.7)	10 (62.5)	18 (54.5)	9 (75.0)	
Exacerbating factors, n (%)					ns
Sleep	2 (3.3)	1 (6.3)	1 (3.0)	0 (0.0)	
Physical activity	13 (21.3)	3 (18.8)	8 (24.2)	2 (16.7)	
Stress	17 (27.9)	3 (18.8)	10 (30.3)	4 (33.3)	
Fatigue	16 (26.2)	2 (12.5)	11 (33.3)	3 (25.0)	
Diet	0 (0.0)	0 (0.0)	0 (0.0)	0 (0.0)	
Hot water	17 (27.9)	5 (31.3)	6 (18.2)	6 (50.0)	
Cold water	0 (0.0)	0 (0.0)	0 (0.0)	0 (0.0)	
Dry air	10 (16.4)	1 (6.3)	8 (24.2)	1 (8.3)	
Sweat	25 (41.0)	7 (43.8)	14 (42.4)	4 (33.3)	
Cold	2 (3.3)	0 (0.0)	1 (3.0)	1 (8.3)	
Warmth	23 (37.7)	5 (31.3)	12 (36.4)	6 (50.0)	
Alleviating factors, n (%)					ns
Sleep	4 (6.6)	0 (0.0)	3 (9.1)	1 (8.3)	
Physical activity	0 (0.0)	0 (0.0)	0 (0.0)	0 (0.0)	
Stress	0 (0.0)	0 (0.0)	0 (0.0)	0 (0.0)	
Fatigue	0 (0.0)	0 (0.0)	0 (0.0)	0 (0.0)	
Diet	0 (0.0)	0 (0.0)	0 (0.0)	0 (0.0)	
Hot water	1 (1.6)	0 (0.0)	1 (3.0)	0 (0.0)	
Cold water	11 (18)	1 (6.3)	5 (15.2)	5 (41.7)	
Dry air	0 (0.0)	0 (0.0)	0 (0.0)	0 (0.0)	
Sweat	0 (0.0)	0 (0.0)	0 (0.0)	0 (0.0)	
Cold	9 (14.8)	2 (12.5)	5 (15.2)	2 (16.7)	
Warmth	1 (1.6)	0 (0.0)	1 (3.0)	0 (0.0)	
Psychological well-being, n (%)					ns
No change	17 (27.9)	4 (25)	9 (27.3)	4 (33.3)	
Decreased mood	13 (21.3)	2 (12.5)	7 (21.2)	4 (33.3)	
Increased mood	1 (1.6)	0 (0.0)	1 (3.0)	0 (0.0)	
Problems with concentration	7 (11.5)	3 (18.8)	2 (6.1)	2 (16.7)	
Anxiety	5 (8.2)	2 (12.5)	0 (0.0)	3 (25.0)	
Anger	1 (1.6)	1 (6.3)	1 (3.0)	0 (0.0)	
Irritation	3 (94.9)	0 (0.0)	2 (6.1)	0 (0.0)	

**Table 3:** Detailed characteristics of dermoscopic findings according to distinguished groups: LPP, FFA, LPP-FFA.

Dermoscopic Features, n (%)	Total (n = 61)	LPP (n = 16)	FFA (n = 33)	LPP—FFA (n = 12)	*p* Value
Absence of openings	61 (100.0)	16 (100.0)	33 (100.0)	12 (100.0)	1.000
White dots	20 (32.8)	6 (37.5)	9 (27.3)	5 (41.7)	0.593
Yellow dots	10 (16.4)	2 (12.5)	6 (18.2)	2 (15.7)	0.880
Red follicular dots	7 (11.5)	4 (25.0)	3 (9.1)	0 (0.0)	0.099
Dilated follicles	7 (11.5)	4 (25.0)	2 (6.1)	1 (8.3)	0.139
Hair diameter diversity	37 (60.7)	7 (43.8)	21 (63.6)	9 (75.0)	0.215
Pili torti	17 (27.9)	2 (12.5)	13 (39.4)	2 (15.7)	0.090
Broken hair	3 (4.9)	1 (6.3)	1 (3.0)	1 (8.3)	0.737
Perifollicular erythema	50 (82.0)	14 (87.5)	25 (75.8)	11 (91.7)	0.376
Perifollicular scaling	43 (70.5)	14 (87.5)	18 (54.5)	11 (91.7)	0.012 *
Perifollicular pigmentation	39 (63.9)	11 (68.8)	19 (57.6)	9 (75.0)	0.502
White halo	24 (39.3)	8 (50.0)	9 (27.3)	7 (58.3)	0.101
Interfollicular white structureless areas	59 (96.7)	16 (100.0)	31 (93.9)	12 (100.0)	0.416
Interfollicular pink structureless areas	51 (83.6)	12 (75.0)	27 (81.8)	12 (100.0)	0.193
Interfollicular white scales	15 (24.6)	5 (31.3)	6 (18.2)	4 (33.3)	0.447
Interfollicular yellow scales	2 (3.3)	2 (12.5)	0 (0.0)	0 (0.0)	0.055
Honeycomb pigmented pattern	12 (19.7)	5 (31.3)	5 (15.2)	2 (16.7)	0.396
Hemorrhages	1 (1.6)	0 (0.0)	0 (0.0)	1 (8.3)	0.125
Erosions	1 (1.6)	0 (0.0)	0 (0.0)	1 (8.3)	0.125

* statistically significant value.

**Table 4:** Relationship between dermoscopic findings and the occurrence, severity at the time of examination (VAS_exam_), and severity of maximum pruritus (VASmax) of the scalp.

	Pruritus, n (%)			VAS_exam_			VAS_max_		
Dermoscopic Finding	Yes	No	*p* Value	Mean ± SD	Median	*p* Value	Mean ± SD	Median	*p* Value
Hair diameter diversity									
yes	32 (52.5)	5 (8.2)		2.4 ± 2.5	2.2		5.5 ± 2.8	5.9	
no	13 (21.3)	11 (18.0)	0.008 *	3.1 ± 2.1	3.2	0.402	6.5 ± 2.8	6.9	0.102
Perifollicular scaling									
Yes	36 (59.0)	7 (11.5)		2.6 ± 2.3	2.3		6.1 ± 2.8	6.6	
no	9 (14.8)	9 (14.8)	0.011 *	2.8 ± 3.1	2.3	0.068	4.5 ± 2.9	5.3	0.005 *
Perifollicular erythema									
yes	39 (63.9)	11 (18.0)		2.5 ± 2.4	2.2		5.8 ± 3.0	6.3	
no	6 (9.8)	5 (8.2)	0.137	3.1 ± 2.3	3.8	0.523	5.6 ± 1.7	6.2	0.205
White halo									
Yes	22 (36.1)	2 (3.3)		2.1 ± 2.5	1.7		5.4 ± 3.0	6.0	
no	23 (37.7)	14 (23.0)	0.016 *	3.1 ± 2.3	3.3	0.599	6.2 ± 2.7	6.6	0.164
Yellow dots									
Yes	10 (16.4)	0 (0.0)		3.0 ± 1.9	2.5		5.6 ± 2.4	6.2	
no	35 (57.4)	16 (26.2)	0.051	2.5 ± 2.5	2.2	0.020 *	5.8 ± 3.0	6.6	0.202
Honeycomb pigment pattern									
Yes	6 (9.8)	6 (9.8)		1.4 ± 2.0	0.4		5.0 ± 2.6	6.0	
no	39 (63.9)	10 (16.4)	0.063	2.8 ± 2.4	2.3	0.012 *	5.9 ± 2.9	6.3	0.037 *

* statistically significant value.

## Data Availability

The datasets generated during and/or analyzed during the current study are available from the corresponding author on reasonable request.
